# Implementation of triple‐scan protocol to evaluate the fit of complete‐arch implant‐supported fixed prostheses

**DOI:** 10.1111/jopr.13830

**Published:** 2024-01-30

**Authors:** Mustafa Borga Donmez, Gülce Çakmak, Martin Schimmel, Burak Yilmaz

**Affiliations:** ^1^ Department of Prosthodontics Faculty of Dentistry Istinye University İstanbul Turkey; ^2^ Department of Reconstructive Dentistry and Gerodontology School of Dental Medicine University of Bern Bern Switzerland; ^3^ Division of Gerodontology and Removable Prosthodontics University Clinics of Dental Medicine University of Geneva Geneva Switzerland; ^4^ Department of Restorative Preventive and Pediatric Dentistry School of Dental Medicine University of Bern Bern Switzerland; ^5^ Division of Restorative and Prosthetic Dentistry The Ohio State University Ohio USA

**Keywords:** complete‐arch, fit, nonmetrology‐grade freeware, triple‐scan

## Abstract

Passive fit is essential for multiple‐unit implant‐supported prostheses. Conventional methods to assess the passivity of complete‐arch implant‐supported prostheses do not allow 3‐dimensional (3D) visualization and quantification of misfit. This report describes the marginal and internal fit evaluation of a complete‐arch implant‐supported prosthesis by using the triple‐scan protocol involving a scanner and a 3D analysis freeware. This technique allows researchers, clinicians, or dental technicians to detect and quantify 3D prosthetic misfit, which may facilitate the preparation for dental appointments and objective measurement of misfit for research studies.

Splinting multiple implants has been believed to help reduce the stress around the implants, distribute occlusal forces, and decrease possible mechanical complications.^1^ A certain advantage of splinting implants is that it is cost‐effective and enables planning of a reduced number of implants.^2^ Four implant‐supported complete‐arch fixed prostheses have become a common treatment modality^3^ given the high survival rates.^4^ However, complications are still possible with ill‐fitting prostheses.^5^

A complete‐arch implant‐supported prosthesis fit is commonly assessed by using the 1‐screw test.^6^ However, 3‐dimensional (3D) misfit might not be detectable if the distortion is on the horizontal plane.^7^ In addition, the 1‐screw test is subjective and relies on the experience and discretion of the observer. With the advancements in digital dental technologies, 3D evaluation of marginal and internal gaps by using 3D analysis software and scanners has become possible.^8^ Marginal and internal gaps of complete‐arch implant‐supported prostheses have been evaluated by researchers or centralized manufacturing facilities with the use of industrial scanners and metrology‐grade 3D analysis software.^1^, ^2^, ^3^, ^5^ However, industrial scanners may not be accessible to dentists and laboratory technicians, and metrology‐grade 3D analysis software programs can be costly as the International Organization for Standardization (ISO) standard 12836 recommends a software program (Geomagic Control X; 3D Systems) that needs to be purchased for dental 3D analyses.^9^ Dental laboratories and clinicians may benefit from the fit evaluation of prostheses on master casts immediately after fabrication to facilitate the preparations for try‐in appointments. Dental researchers may also benefit from the ability to use readily available equipment for such analyses. However, alternative techniques are necessary to perform the chairside evaluation of complete‐arch implant‐supported prosthesis fit due to the abovementioned limitations with industrial and metrology‐grade hardware and software programs.

Recently, a 3D analysis freeware program (Medit Link; Medit) has become accessible for clinicians and dental technicians,^10^, ^11^ and has been shown to perform similarly to the ISO‐recommended metrology‐grade 3D analysis software program while evaluating the fabrication trueness of complete‐arch implant‐supported frameworks.^12^ This freeware program and scans from laboratory or intraoral scanners enable the measurement of marginal and internal gaps of complete‐arch implant‐supported prostheses on master casts immediately after fabrication.^12^ This report describes a technique to measure marginal and internal gaps of a complete‐arch implant‐supported prosthesis supported with four implants by using the triple‐scan protocol.^13^ This method relies on the superimposition of three separate scans that are of the prosthesis, model, and when the prosthesis is tightened on the implants or abutments in an implant‐supported situation. The use of the triple scan protocol has been demonstrated for single crowns and fixed partial dentures^14^, ^15^, ^16^; however, this protocol has not been detailed in the literature, step by step, for the analysis of complete‐arch implant‐supported prosthesis fit. Therefore, the present technique can help present the details of this protocol.

## TECHNIQUE

**FIGURE 1:**
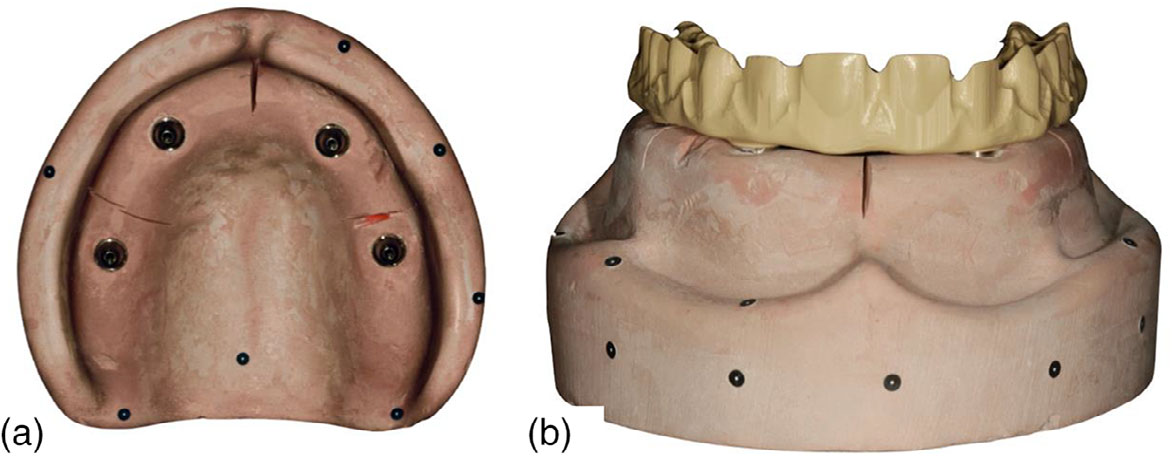
Occlusal aspect of complete‐arch implant‐supported maxillary cast. (a) Without prosthesis; (b) With prosthesis.

**FIGURE 2:**
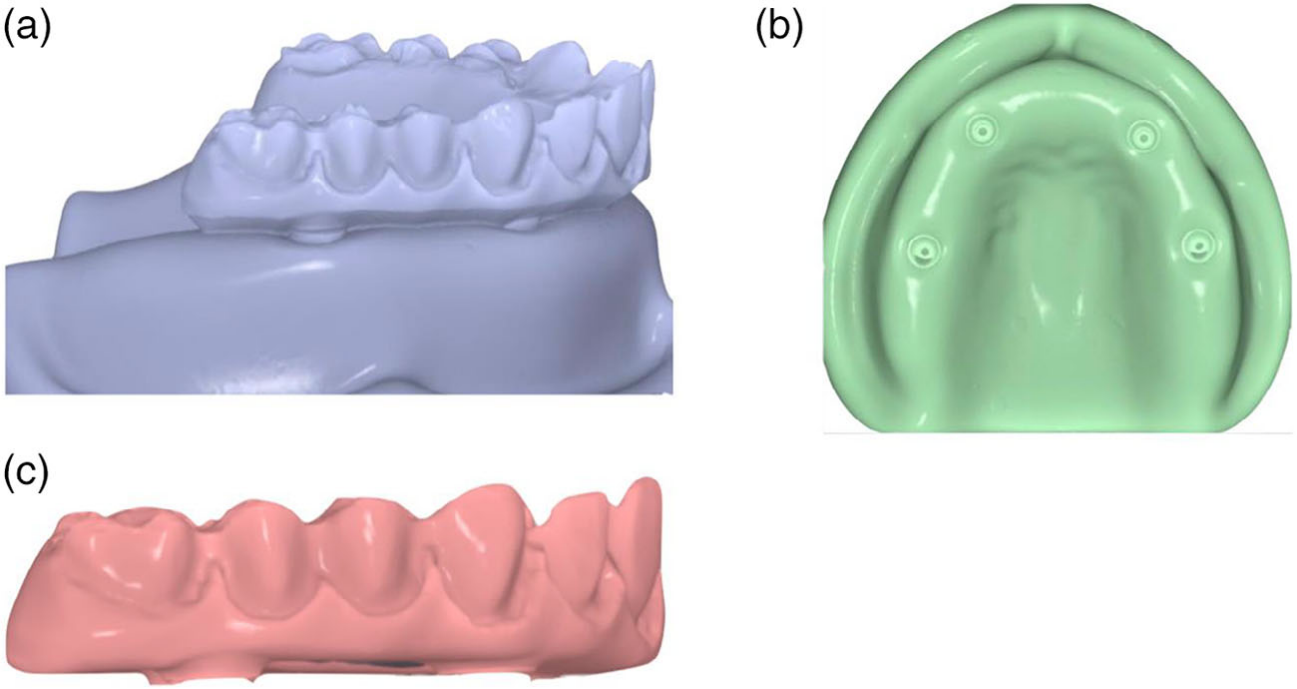
Generated STL files. (a) Key scan STL; (b) Model STL; (c) Prosthesis STL; STL, Standard tessellation language.

**FIGURE 3:**
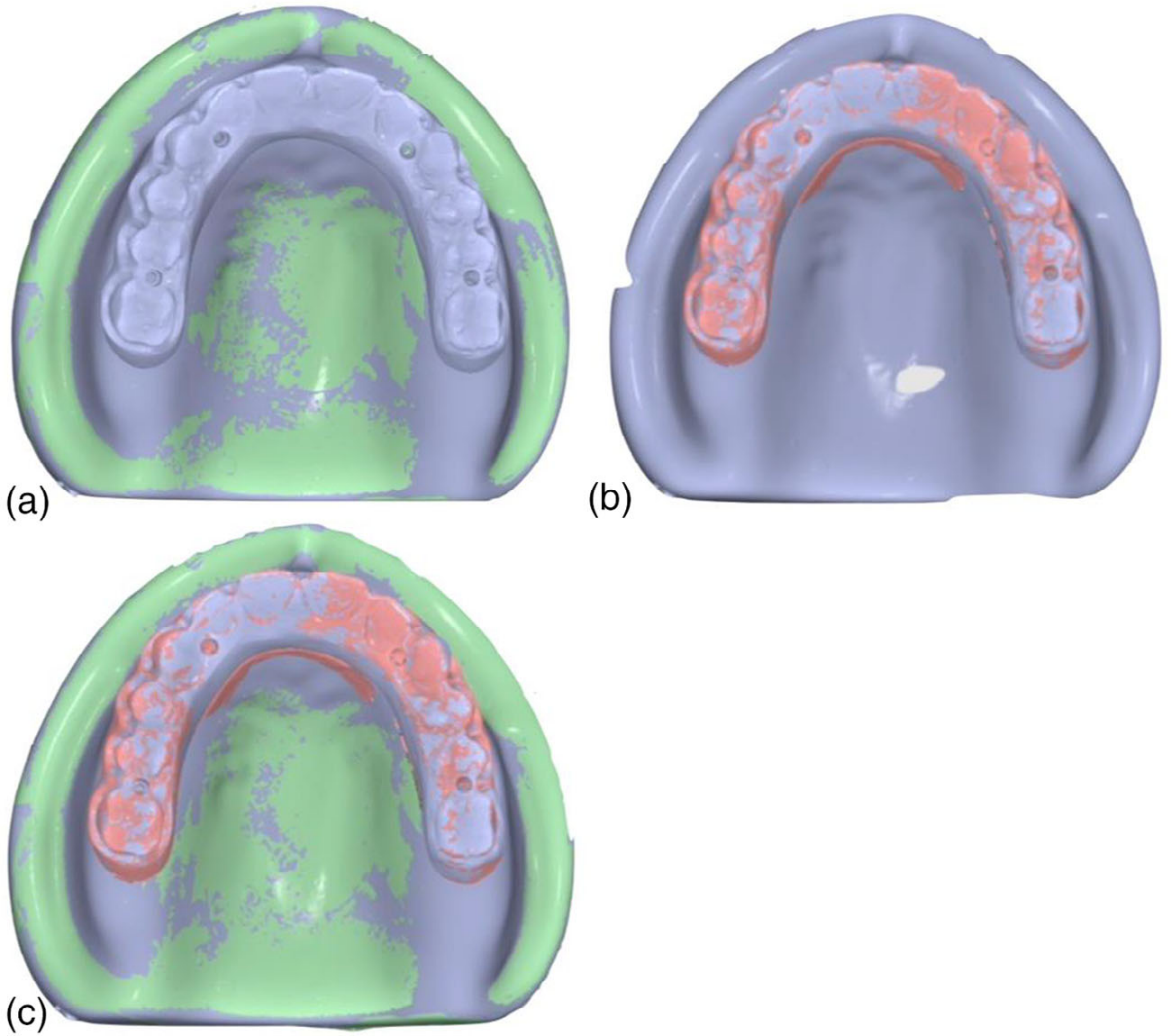
Superimposition of STL files. (a) Key scan STL over model STL; (b) Prosthesis STL over key scan STL; (c) Model STL and prosthesis STL over key scan STL; STL, Standard tessellation language.

**FIGURE 4:**
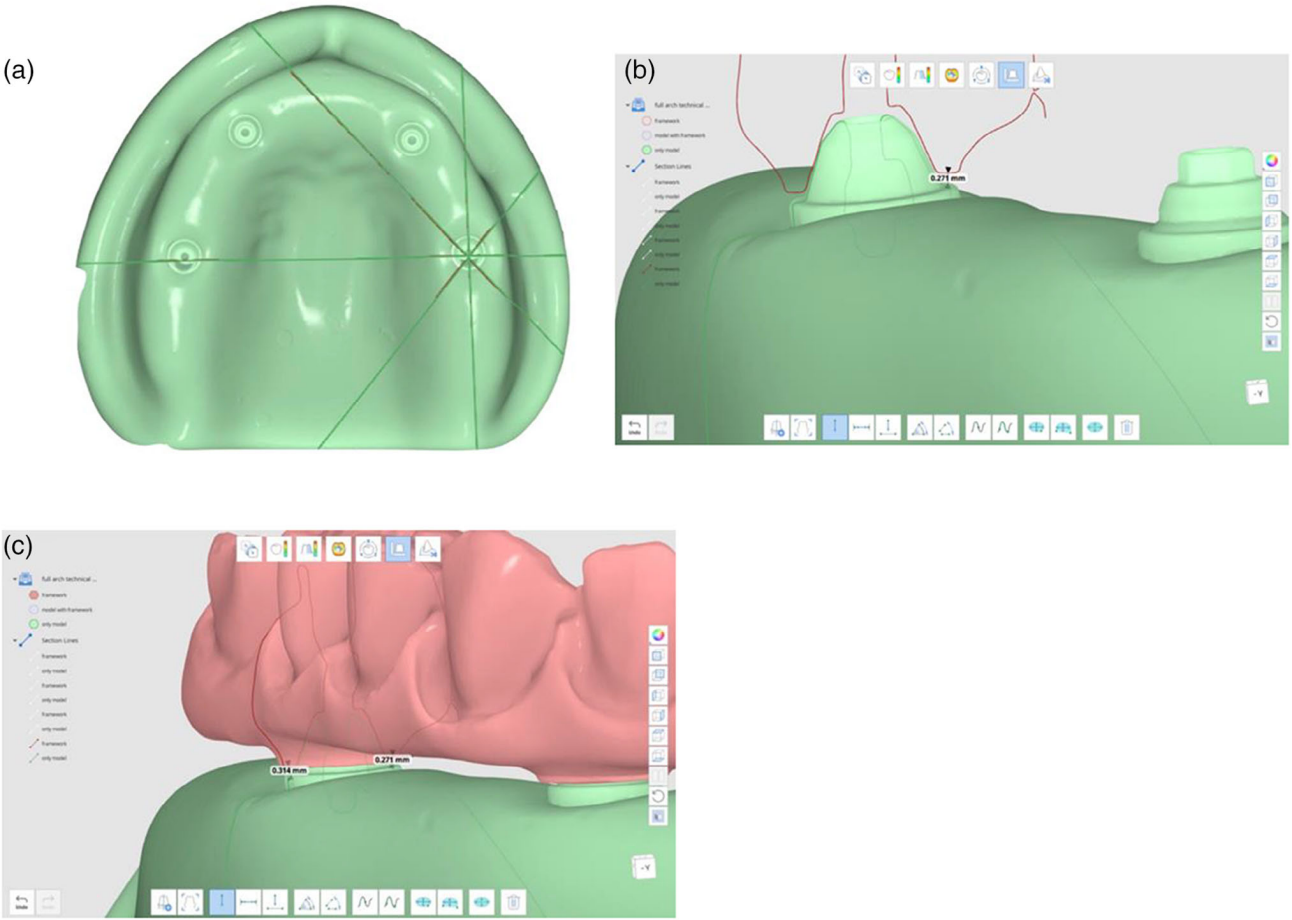
Fit evaluation. (a) Planes generated for evaluation; (b) Cross‐sectional view of prosthesis on model; (c) Distance between corresponding points on model and prosthesis.

1. Place the complete‐arch implant‐supported prosthesis on the master cast (Figure 1a) with multi‐unit abutments and tighten the prosthetic screws on the left molar abutment (terminal location) and right canine by using a hand screwdriver (Figure 1b).
2. Using a calibrated torque wrench, tighten the prosthetic screw on the terminal abutment to 15 Ncm and unscrew the prosthetic screw at the right canine.
3. Scan the entire cast and the complete‐arch implant‐supported prosthesis by using an intraoral scanner or a laboratory scanner to generate a key scan standard tessellation language (STL) file (Figure 2a).
4. Unscrew the prosthetic screw on the terminal abutment and scan the entire model by using the same scanner without the prosthesis to generate a model STL file (Figure 2b).
5. Scan the entire prosthesis to generate a prosthesis STL file (Figure 2c). For those situations, in which a laboratory scanner is used, first scan the occlusal and then the gingival aspects of the prosthesis and then stitch these two scans to generate the prosthesis STL file. This step can be finished at once when an intraoral scanner is used. When a highly reflective material such as titanium is scanned, use an anti‐reflective spray to facilitate the scan.
6. Import all STLs into a 3D analysis freeware program (Medit Link; Medit). By using the “design” tool of the freeware program and best‐fit algorithm, initially superimpose the key scan STL (target data) over the model STL (reference data) by selecting areas three points other than the prosthesis to avoid deviations that may have occurred during alignment (Figure 3a). Then use the “alignment with selected areas” feature of the freeware program for further alignment.
7. Superimpose prosthesis STL (target data) over the key scan STL (reference data) with the same methodology (Figure 3b). Finally, select key scan and prosthesis STLs as target data and model STL as reference data for the software program to superimpose all STLs automatically. These consecutive superimpositions will transfer all STL files to the same coordinate system for marginal gap evaluation (Figure 3c).
8. Deselect or delete key scan STL after superimpositions to evaluate the adaptation of the prosthesis on the model. Viewing the occlusal aspect, generate four sectional planes on each abutment position (Figure 4a) other than the terminal abutment by using the “create sections” feature of the software program to measure the gaps between the prosthesis STL and the model STL at the abutment‐prosthesis interface (Figure 4b).
9. Measure the closest distance between corresponding points on the abutment and the prosthesis generated by sectional planes by using the “measure distance by one point” feature of the software program (Figure 4c). As many points desired as possible may be selected for measurements.

## DISCUSSION

The primary advantage of the presented technique is that it facilitates prosthesis fit assessment after fabrication and before a try‐in appointment. STL files either from a laboratory scanner or an intraoral scanner can be used, which enables the technique's applicability in a dental laboratory or a clinic. Evaluation of prosthesis fit, particularly in the dental laboratory, can enable the detection of hard‐to‐detect misfits in 3D and prepare both the dental technician and clinician for upcoming steps, particularly for those situations where a remake is necessary. However, it should be emphasized that digitally measured marginal gap values should not be interpreted as a direct indicator of passivity and should be accounted as a possible indicator. The misfit thresholds for implant‐supported prostheses have been reported to be between 30 and 160 µm vertically and 150 µm horizontally for mechanical complications. These values were broader when biological complications were considered with 1 mm of vertical and 345 µm of horizontal misfit.^17^ However, these values were mostly derived from in vitro studies and universally accepted misfit values for implant‐supported prosthesis are not certain.

Previous studies have reported that the nonmetrology‐grade 3D analysis freeware program used in the present report performed similarly to metrology‐grade software in terms of accuracy while analyzing single crowns,^11^ single implant scans,^10^ and complete‐arch implant‐supported frameworks.^12^ However, Dede et al.^12^ also concluded that the source of the STL file may lead to significant differences between the nonmetrology‐grade freeware program and the ISO‐recommended metrology‐grade software program while evaluating marginal deviations of the complete‐arch implant‐supported frameworks. Nevertheless, this nonmetrology‐grade freeware program is not the only option to implement this technique. A trial version of a metrology‐grade freeware program (GOM Inspect; GOM GmbH) has been used in some previous studies for marginal gap evaluation of complete‐arch implant‐supported prostheses.^3^, ^5^ This freeware program allows a standardized selection of points for superimpositions through a coordinate selection feature,^10^ which may be efficient for researchers. When combined with an accurate software program that has high inter‐rater reliability between different operators, this technique may become a routine in dental research for misfit evaluation and in dental clinics or laboratories to facilitate efficient use of time for chairside applications and reduce the probability of complications related with misfit.

Laboratory scanners were reported to have higher accuracy than intraoral scanners.^8^ However, the accuracy of intraoral scanners has improved substantially since they were first introduced,^18^ and scan inaccuracy as low as 7 µm has been reported.^19^ Another advantage of using an intraoral scanner is the possibility of completing the entire prosthesis scan in one continuous motion. While using a laboratory scanner, separate scans of the occlusal and gingival aspects of the prosthesis need to be scanned and then digitally stitched with the scanner's algorithm. This additional step may lead to amplified deviations. A recent study has also shown that intraoral scanners may be used to evaluate the fabrication trueness of complete‐arch implant‐supported frameworks when fabricated in polyetheretherketone.^20^ In addition, even though none of the scans of the tested intraoral or laboratory scanners had similar deviations to those of the scans of an industrial‐grade blue light optical scanner, the maximum estimated mean differences between the scans was 31.35 µm overall and 53.90 µm for marginal surface deviations. These results were supported by those of another recent study, in which the maximum raw mean difference between the scans of intraoral scanners and an industrial‐grade blue light optical scanner was 37.33 µm and the maximum raw confidence interval value of estimated differences was 47.88 µm when fabrication trueness of titanium complete‐arch implant‐supported frameworks were evaluated.^21^ Even though those studies^20^, ^21^ did not involve marginal fit analysis, the mean differences reported are relatively low for a large‐size framework like a complete‐arch prosthesis and intraoral scanners may be suitable for the fit analysis with the triple‐scan protocol after in vitro validation.

The reported technique has the abovementioned advantages, and the nonmetrology‐grade freeware program has a user‐friendly interface. However, 3D analysis software programs involve a learning curve, which should be taken into consideration. Also, digital trueness analyses should be accounted as an assessment auxiliary to intraoral misfit evaluation, and the try‐in appointment should not be eliminated even when small gaps are measured in the digital triple scan fit analysis. In addition, this digital analysis method might not completely replicate the clinical situation between the framework and the abutment, where there is physical contact between these components.^22^ Clinicians should calibrate themselves with radiographs and 1‐screw tests to make a judgment on the passivity of a prosthesis based on the results of the internal and marginal gap values measured by using this technique.

## CONCLUSION

The presented report describes a technique to evaluate the marginal and internal gaps of complete‐arch implant‐supported fixed prostheses by using the triple‐scan protocol and nonmetrology‐grade or metrology‐grade 3D analysis programs. This technique enables quantitative gap evaluation for research studies and facilitates the evaluation of the prosthesis fit prior to the clinical appointment, increasing the clinician's or dental technician's awareness of possible misfit.

## CONFLICT OF INTEREST STATEMENT

The authors declare no conflict of interest. The authors do not have any financial interest in the companies whose materials are included in this article.
